# Effectiveness of Acupuncture for Infertility in Patients with Polycystic Ovary Syndrome: A Systematic Review and Network Meta-Analysis

**DOI:** 10.2174/0118715303297819240826065755

**Published:** 2024-09-20

**Authors:** Yumi Wu, QiWei Xiao, ShouDong Wang, HuanFang Xu, YiGong Fang

**Affiliations:** 1 Institute of Acupuncture and Moxibustion of China Academy of Chinese Medical Sciences, Beijing, China;; 2 The Out-Patient Department of TCM of China Academy of Chinese Medical Sciences, Beijing, China;; 3 Acupuncture and Moxibustion Hospital of China Academy of Chinese Medical Sciences, Beijing, China

**Keywords:** Acupuncture, polycystic ovary syndrome, infertility, clomiphene citrate, letrozole, estrogens

## Abstract

**Objective:**

This study employs a network meta-analysis method to investigate the clinical effectiveness of acupuncture in patients with polycystic ovary syndrome (PCOS) experiencing infertility.

**Methods:**

Prospective randomized controlled trials (RCTs) of clomiphene citrate (CC) and letrozole (LE) combined with acupuncture in PCOS infertility patients were identified through computerized searches in databases including PubMed, Web of Science, Embase, Cochrane Library, China National Knowledge Infrastructure (CNKI), Wanfang Data, and Chongqing VIP Database. The search period was set from inception until August 1, 2023, with no language restrictions. Two researchers screened articles, extracted data, and independently assessed the risk of bias in eligible trials. Data were analyzed and visualized using the R software gemtc package. With patients with medication treatment only set as controls, a meta-analysis was performed to investigate the difference in the pregnancy outcomes of the PCOS patients following medication amalgamated with different acupuncture treatments, namely, manual acupuncture (MA), electroacupuncture (EA), and warm acupuncture (WA).

**Results:**

The serum concentrations of follicle-stimulating hormone (FSH) did not exhibit significant changes following acupuncture treatments. Notably, acupuncture-based medication treatment significantly reduced serum levels of luteinizing hormone (LH) and elevated the testosterone (T) concentrations of patients when compared to medication treatment alone. Patients also showed significantly escalated serum estradiol (E2) levels after receiving CC integrated with acupuncture than those given monotherapy of CC. The combined regimen of medication and acupuncture appeared to improve the pregnancy outcomes compared to the monotherapy of medication, as evidenced by the significantly increased success rate of pregnancy. Furthermore, the treatment combination of CC plus WA and LE plus MA yielded the highest probability of achieving the best pregnancy outcomes.

**Conclusion:**

For PCOS infertility patients, acupuncture, as a complementary treatment to CC and LE, holds advantages in improving reproductive hormone levels and enhancing pregnancy success rates. The highest probability of achieving the best pregnancy outcomes is associated with the treatment combination of CC with WA and LE with MA.

## 1 INTRODUCTION

Polycystic Ovary Syndrome (PCOS) is a disorder caused by disruptions in the endocrine system, closely associated with aberrant serum concentrations of luteinizing hormone (LH), follicle-stimulating hormone (FSH), androgens, estrogens, and progesterone [1]. PCOS exerts a wide-ranging influence on women, encompassing reproduction and pregnancy-related challenges, but also extending to factors such as insulin resistance, type 2 diabetes, cardiovascular conditions, and psychological well-being [2-4]. PCOS is characterized by anovulation, hyperandrogenism, and polycystic ovarian morphology, with a prevalence of approximately 6-9% in premenopausal women [5]. It stands as a major cause of anovulatory infertility, with approximately 70-80% of women with PCOS experiencing infertility [6].

To date, no unanimity has been established for the definitive treatment for PCOS, and current approaches focus on symptomatic treatment and long-term health management, such as oral contraceptives, surgical interventions, and assisted reproductive technologies (ART) [7]. The treatment objectives vary from inducing ovulation and achieving pregnancy for PCOS patients desiring fertility [8, 9], to restoring menstrual cycles, treating acne, managing weight, and preventing complications such as diabetes, hypertension, hyperlipidemia, cardiovascular diseases, and endometrial cancer for those without fertility needs [10]. Mesenchymal stem cell-derived exosomes have significant potential in the treatment of PCOS, but their applications are not yet widespread [11]. The first-line pharmacotherapy for anovulatory infertility in PCOS women is selective estrogen receptor modulators, such as oral clomiphene citrate (CC). However, the clomiphene citrate regimen has been reported to be underpowered in up to 40% of affected women [12]. Hence, suitable alternative therapies have been the subject of significant discussion in PCOS management.

Acupuncture, an integral part of traditional Chinese medicine (TCM), has gained popularity in the treatment of various diseases due to its convenience and low incidence of adverse effects. Research investigating the use of acupuncture in PCOS treatment has been expanding, indicating a potential beneficial effect on ovulation and fertility [13]. Nevertheless, there has been limited research on the application of acupuncture to address reproductive issues in PCOS. Many studies suffer from methodological limitations, inadequate study design, and a shortage of reliable outcome measures and diagnostic criteria, which complicate the interpretation of findings.

To this end, we embark on a network meta-analysis to compare the benefits of different treatment methods based on acupuncture on fertility in PCOS patients.

## 2 MATERIALS AND METHODS

### 2.1 Search Strategy

Databases including PubMed, Web of Science, Embase, Cochrane Library, China National Knowledge Infrastructure (CNKI), Wanfang Data, and Chongqing VIP Database were searched. The search time was from database establishment to August 2023, with no language restrictions. The search strategy employed combinations of terms such as “Polycystic Ovary Syndrome,” “PCOS,” “Acupuncture,” and “Electroacupuncture.” As an example, the search strategy for PubMed is outlined in Table **1**.

When conducting systematic reviews and network meta-analyses, selection of databases such as PubMed, Web of Science, Embase, Cochrane Library, China National Knowledge Infrastructure (CNKI), Wanfang Data, and Chongqing VIP Database (VIP) as retrieval sources is based on the following reasons:

1. Authority and comprehensiveness: These databases are well-known academic databases both domestically and internationally, covering high-quality academic literature in various fields such as medicine, life sciences, social sciences, and natural sciences. Among them, PubMed, Web of Science, and Embase are highly authoritative in the biomedical field, indexing numerous journal articles and conference papers. Cochrane Library focuses specifically on evidence-based medicine research, providing high-quality evidence support.
2. Comprehensive literature coverage: These databases have their own characteristics in terms of literature inclusion, complementing each other. For example, PubMed mainly indexes literature from the MEDLINE database, while Web of Science includes authoritative journals from multiple disciplines through its strict selection mechanism. Embase, on the other hand, focuses more on literature from Europe and Asia. At the same time, CNKI, Wanfang Data, and VIP Database primarily include Chinese literature, providing important resources for systematic reviews on Chinese research.
3. Meeting specific research needs: Different databases have differences in terms of literature types, research fields, and inclusion time, thereby meeting the requirements of different types of research. For example, network meta-analysis requires comparing the effects of multiple interventions, and these databases provide rich data support, enabling researchers to gain a comprehensive understanding of the differences in intervention effects.
4. Ensuring research reliability: Using multiple databases for retrieval can reduce the risk of missing relevant studies, thereby enhancing the reliability and accuracy of the research. Additionally, these databases have strict quality control mechanisms for literature, ensuring the quality of the retrieved articles.

### 2.2 Literature Selection Criteria

Following the PICOS principle, the inclusion criteria were as follows: Population (P): Females, specifically those classified as PICO infertility patients; Intervention (I): At least one of the following interventions - Manual Acupuncture (MA), Warm Acupuncture (WA), Electroacupuncture (EA), as well as at least one of the medications Clomiphene Citrate (CC) and Letrozole (LE); Control (C) included sole use of CC or LE; Outcome (O): Primary outcomes included pregnancy rate, and secondary outcomes comprised FSH, LH, E2, T; Study Design (S) included Randomized Controlled Trials (RCTs). Studies involving concomitant TCM treatments, simultaneous use of multiple acupuncture methods, and those with fewer than 10 participants were excluded.

### 2.3 Data Extraction

Two independent researchers utilized a pre-defined Excel format to extract data from the included studies. This encompassed the first author's name, publication date, sample size, subject age, intervention methods, treatment duration, outcome indicators, adverse events, methods of randomization, allocation concealment, blinding, and other pertinent information. Any discrepancies encountered during the data extraction process were resolved through discussion with a third researcher.

### 2.4 Literature Quality Assessment

Two evaluators assessed the methodological quality of all RCTs using the Cochrane Risk of Bias Assessment Tool, encompassing seven domains: (1) random sequence generation, (2) allocation concealment, (3) blinding of participants and personnel, (4) blinding of outcome assessment, (5) incomplete outcome data, (6) selective reporting, and (7) other biases. These studies were classified as “low risk,” “high risk,” or “unclear risk.” Any discrepancies were resolved through consensus or adjudication by a third researcher.

### 2.5 Statistical Analysis

The meta-analysis was conducted using the R language and the meta package. Risk Ratios (RR) and 95% Confidence Intervals (CI) were utilized for analyzing binary variables, while Standardized Mean Differences (SMD) and 95% CI were used for continuous variables. The I2 test was employed to assess heterogeneity among studies; an I2 of 0% indicates no observed heterogeneity, with higher values indicating greater heterogeneity. In this study, I2>50% was considered to indicate substantial heterogeneity, requiring subgroup analysis or analysis using a random-effects model. When heterogeneity still persisted among included studies after subgroup analysis, sensitivity analysis was conducted by excluding studies with a high risk of bias (e.g., small sample size, methodological flaws, and missing data) to re-perform the meta-analysis, thereby ensuring the robustness of the analysis results. Publication bias was assessed by generating a funnel plot and conducting Egger's test. A significance level of α=0.05 was used to determine statistical significance.

## 3 RESULTS

### 3.1 Literature Selection Results

Following the initial computerized search and import into Endnote, a total of 632 articles were retrieved. After excluding 212 duplicate articles, the titles and abstracts of the remaining 520 articles were screened. Upon reviewing the titles and abstracts, 501 articles were excluded, with 61 being non-clinical studies such as reviews, conference reports, and commentaries, 248 not meeting the specified intervention criteria, and 192 unrelated to the research topic. The full texts of the remaining 19 articles were examined, culminating in the exclusion of 2 articles lacking specified outcome measures and 6 lacking specified intervention methods. Ultimately, 11 studies were included in the analysis. The flowchart illustrating the literature selection process is depicted in Fig. (**1**).

### 3.2 Basic Information of Included Studies

A total of 11 studies were ultimately included, involving 1517 participants. Among them, there were 752 participants in the control group and 735 participants in the study group. Regarding the intervention approaches in the control group, 8 studies applied CC treatment, while 3 studies utilized LE treatment. As for the research group, 4 studies employed CC plus WA, 2 studies utilized CC plus MA, 2 studies used CC plus EA, 1 study implemented LE plus WA, and 2 studies utilized LE plus MA treatment. In terms of outcome measures, all 8 studies presented Fugl-Meyer Assessment results, and 4 studies included modified Barthel Index results. The essential details of the incorporated studies can be found in Table **2**. Fig. (**2**) displays the quality assessment of the included studies.

### 3.3 Meta-Analysis

A meta-analysis was carried out to compare the effects of medication treatment and acupuncture-based medication on reproductive hormones and pregnancy outcomes. The control group interventions involved the use of either CC or LE treatment, while the study group received any one of the following: CC plus MA, CC plus WA, CC plus EA, LE plus MA, or LE plus WA.

#### 3.3.1 FSH Outcomes

Among the included studies, 9 reported FSH outcomes, with 6 studies utilizing acupuncture-based CC treatment and 3 studies employing acupuncture-based LE treatment. Heterogeneity analysis indicated significant heterogeneity among the studies, warranting the use of a random-effects model for analysis. The meta-analysis results showed that the Standardized Mean Difference (SMD) for the FSH indicator between medication treatment and acupuncture-based medication was 0.23 (95% CI: -0.08, 0.54); between CC treatment and acupuncture-based CC treatment, it was 0.29 (95% CI: -0.12, 0.70); and between LE treatment and acupuncture-based LE treatment, it was 0.29 (95% CI: -0.41, 0.66). The forest plot for the FSH across different treatment modalities is shown in Fig. (**3**), indicating no statistically significant differences in FSH levels.

#### 3.3.2 LH Outcomes

Among the included studies, a total of 9 reported LH outcomes, with 6 studies utilizing acupuncture-based CC treatment and 3 studies employing acupuncture-based LE treatment. Heterogeneity analysis indicated significant heterogeneity among the studies, prompting the use of a random-effects model for analysis. The meta-analysis results showed that the SMD for the LH between medication treatment and acupuncture-based medication was 0.96 (95% CI: 0.38, 1.53); between CC treatment and acupuncture-based CC treatment, it was 0.46 (95% CI: 0.14, 0.77); and between LE treatment and acupuncture-based LE treatment, it was 1.98 (95% CI: 1.09, 2.87). The forest plot for the LH across different treatment modalities is shown in Fig. (**4**), indicating that acupuncture-based Western medicine treatment significantly reduces LH levels compared to sole medication treatment.

#### 3.3.3 E2 Outcomes

Among the included studies, a total of 8 reported E2 outcomes, with 5 studies utilizing acupuncture-based CC treatment and 3 studies employing acupuncture-based LE treatment. Heterogeneity analysis indicated significant heterogeneity among the studies, prompting the use of a random-effects model for analysis. The meta-analysis results showed that the SMD for the E2 indicator between medication treatment and acupuncture-based medication was -0.49 (95% CI: -1.35, 0.38); between CC treatment and acupuncture-based CC treatment, it was -0.99 (95% CI: -1.29, -0.69); and between LE treatment and acupuncture-based LE treatment, it was 0.34 (95% CI: -1.84, 2.52). The forest plot for the E2 across different treatment modalities is shown in Fig. (**5**), indicating that acupuncture-based CC treatment significantly increases E2 levels compared to sole Western medicine treatment.

#### 3.3.4 Serum Levels of T

Among the included studies, a total of 7 reported serum levels of T, with 4 studies utilizing acupuncture-based CC treatment and 3 studies employing acupuncture-based LE treatment. Heterogeneity analysis indicated significant heterogeneity among the studies, warranting the use of a random-effects model for analysis. The meta-analysis results showed that the SMD for the T between medication treatment and acupuncture-based medication was 1.02 (95% CI: 0.31, 1.73); between CC treatment and acupuncture-based CC treatment, it was 0.51 (95% CI: -0.02, 1.03); and between LE treatment and acupuncture-based LE treatment, it was 2.03 (95% CI: 1.61, 2.44). The forest plot for the T across different treatment modalities is shown in Fig. (**6**), indicating that acupuncture-based Western medicine treatment significantly reduces T levels compared to sole medication treatment.

#### 3.3.5 Pregnancy Outcomes

Among the included studies, a total of 9 reported pregnancy outcomes, with 6 studies utilizing acupuncture-based CC treatment and 3 studies employing acupuncture-based LE treatment. Heterogeneity analysis indicated significant heterogeneity among the studies, prompting the use of a random-effects model for analysis. The meta-analysis results showed that the Odds Ratio (OR) for pregnancy outcome between medication treatment and acupuncture-based medication was 1.82 (95% CI: 1.43, 2.33); between CC treatment and acupuncture-based CC treatment, it was 1.59 (95% CI: 1.20, 2.10); and between LE treatment and acupuncture-based LE treatment, it was 2.93 (95% CI: 1.74, 4.95). The forest plot for pregnancy outcomes across different treatment modalities is shown in Fig. (**7**), indicating that acupuncture-based Western medicine treatment significantly increases pregnancy rates compared to medication treatment.

The publication bias funnel plots for LH, T, and pregnancy outcomes among different studies are shown in Fig. (**8**). Significant publication bias was observed in the literature related to LH, T, and pregnancy outcomes, demonstrating significant asymmetry. The potential reason for this could be attributed to differences in intervention methods.

### 3.4 Network Meta-Analysis

The meta-analysis results corroborated a significant increase in pregnancy success rates with acupuncture-based CC and LE treatments. To further explore the differences in pregnancy outcomes among different treatment modalities, treatments were categorized into seven groups: CC, LE, CC plus MA, CC plus WA, CC plus EA, LE plus MA, and LE plus WA. The network relationship diagram among different treatment modalities is shown in Fig. (**9**).

The results indicated that the pregnancy success rate of CC plus WA was significantly higher than CC alone. The pregnancy outcomes were similar between CC plus WA, CC plus MA, and CC plus EA. The probability of achieving the best pregnancy outcome was the highest for CC plus WA (73.4%), followed by CC plus MA (19.7%), and CC alone had the lowest probability (0.1%).

**Fig. (10):**
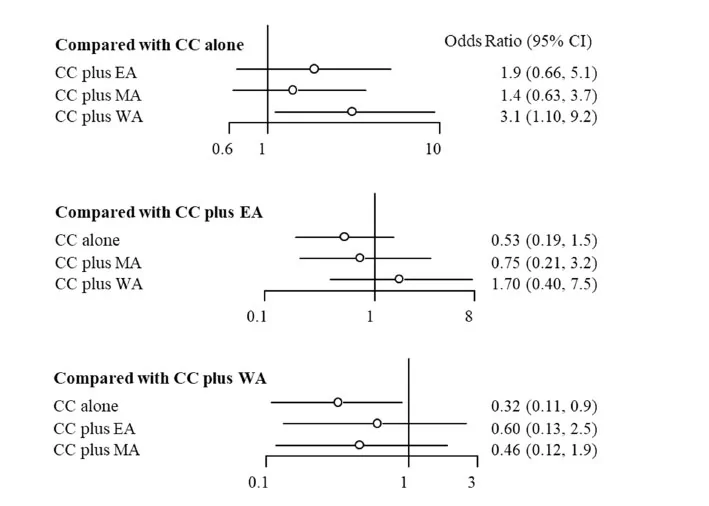
Comparison of pregnancy outcomes with clomiphene citrate combined with different acupuncture methods.

**Fig. (11):**
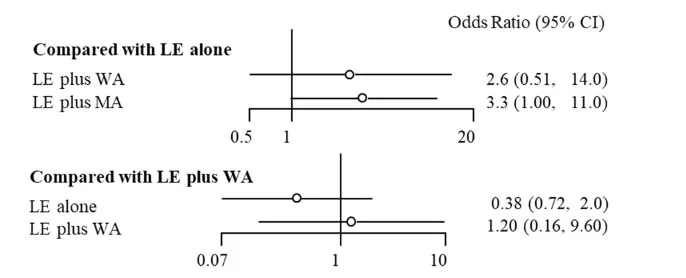
Comparison of pregnancy outcomes with letrozole combined with different acupuncture methods.

The findings suggested that the pregnancy success rate of LE plus MA was significantly higher than that of letrozole alone. No significant differences in pregnancy outcomes were observed between LE plus WA and LE plus MA. The likelihood of achieving the most favorable pregnancy outcome was the highest for LE plus MA (60.8%), followed by LE plus WA (39.5%), while letrozole alone had the lowest likelihood (0.4%) [14-24] (Figs. **10** and **11**).

## 4 DISCUSSION

Currently, clinical literature on acupuncture reports satisfactory therapeutic outcomes, with some studies suggesting the effectiveness of acupuncture in promoting ovulation and reducing BMI and T-values [25, 26]. Additionally, certain studies have reported positive effects of acupuncture on improving patients' basal body temperature [27]. However, there is a lack of consensus regarding the efficacy targets and clear elucidation of the mechanisms of acupuncture treatment for PCOS. Therefore, the effectiveness of acupuncture in treating PCOS needs to be validated using evidence-based medicine methods.

This study included 9 RCTs to evaluate the effectiveness of acupuncture-based treatments for infertility in patients with PCOS. In contrast to earlier meta-analyses, this study omitted research involving acupuncture combined with TCM treatment. This exclusion helped minimize variables that might influence the assessment of effectiveness, resulting in more reliable outcomes [25, 26]. The study found that compared to using CC or LE alone, the integration of acupuncture significantly reduced serum LH and T levels of POCS patients. Additionally, compared to using CC alone, concomitant administration of acupuncture significantly increased serum E2 levels. Furthermore, acupuncture-based medication, when compared to medication alone, led to an increased pregnancy success rate. The differences in FSH indicators among different treatment modalities did not reach statistical significance, possibly due to variations in the quality of each study.

Infertility caused by PCOS is closely related to the disruption of the hypothalamic-pituitary-ovary/adrenal axis, leading to increased LH release, decreased FSH levels, a reversed LH/FSH ratio, and subsequently, ovulation cessation and infertility [27]. The primary approach for inducing ovulation in PCOS-infertility patients is lifestyle intervention, with the frequently prescribed medications being CC and LE [28]. Clomiphene citrate, a commonly used clinical ovulation-inducing drug, competes with endogenous estrogen at the hypothalamic-pituitary level, inhibiting estrogen-negative feedback, adjusting the secretion ratio of LH and FSH, and directly stimulating ovarian synthesis and secretion of estrogen. In low doses, it can activate estrogen receptors in the hypothalamus and pituitary, reducing the secretion of gonadotropins. In women with intact pituitary and ovaries, it can elevate serum FSH levels, thereby prompting ovulation [29, 30]. Letrozole, a non-steroidal aromatase inhibitor, widely used in hormonal adjuvant therapy for postmenopausal breast cancer patients, is administered for the treatment of PCOS infertility. It holds the capability to inhibit the production of estrogen, diminishing its inhibitory effect on the pituitary gland. This alleviates the suppressive influence of estrogen on the gonadal axis, ultimately leading to enhanced follicle development and ovulation [31, 32]. However, clomiphene citrate may lead to symptoms such as ovarian enlargement, nausea, vomiting, breast discomfort, visual symptoms, insomnia, headache, dizziness, increased urinary frequency, heavy menstrual flow, depression, and fatigue. Contrarily, letrozole does not interfere with the normal development of the endometrium and only triggers mild side effects, such as hot flashes, joint pain, palpitations, nausea, and fatigue [33].

Acupuncture involves inserting needles into specific points on the skin and underlying muscle tissue, known as acupoints. Currently, acupuncture features a high safety profile and serves as a common treatment method or adjunctive treatment of various diseases [34]. Acupuncture is classified as manual acupuncture, electroacupuncture, and warming acupuncture. Manual acupuncture entails stimulating the needle through manual rotation or vertical movement. Electroacupuncture involves applying an electric field and conducting a current between two needles to elicit stimulation. Warm acupuncture includes heating the needle handle using techniques such as moxibustion and integrating it with acupuncture to address various medical conditions. The neuroendocrine mechanism of acupuncture has been validated in reproductive medicine. On one aspect, it can modulate the hypothalamic-pituitary-ovarian axis, facilitating regular ovulation and the reinstatement of menstrual cycles [13]. Simultaneously, it can influence the local function of the ovaries by impacting the expression of cytokines. This leads to the restoration of ovarian function, enabling normal ovulation, and ultimately enhancing pregnancy rates [35, 36]. Acupuncture is widely used in the treatment of PCOS, but a recent large-scale deconstructive trial negated the reproductive and metabolic benefits of acupuncture for PCOS patients. This has caused considerable controversy since its publication [24]. Nonetheless, this study's conclusion is limited as it only assessed the effectiveness of acupuncture combined with MA. This research affirms the impact of acupuncture in conjunction with CC and LE on enhancing reproductive hormones and pregnancy rates. Furthermore, it scrutinizes the distinctions in pregnancy rates across various acupuncture approaches. The findings suggest that the likelihood of attaining the most favorable pregnancy outcome is highest with CC combined with WA, as well as with LE integrated with MA. Nevertheless, additional verification is needed to pinpoint the exact reasons behind this trend. However, there are uncertainties regarding the applicability of acupuncture in patients with PCOS who have concomitant organic lesions involving the uterus and adnexa. Adverse events such as syncope or back spasms before acupuncture needling may also occur.

Systematic reviews and network meta-analyses are commonly used research methods in the medical field, aiming to systematically assess the effects of multiple interventions and compare them. In the case of acupuncture treatment for PCOS-related infertility, employing these methods would not only focus on comparing interventions. A systematic review conducts comprehensive literature searches, screening, quality assessment, and data analysis of all relevant studies on acupuncture treatment for PCOS-related infertility, aiming to understand the overall effectiveness of acupuncture. During this process, researchers pay attention to different acupuncture treatment protocols, combined use of acupuncture with other treatment modalities, and the efficacy of acupuncture in different patient populations.

Network meta-analysis expands the scope of systematic reviews by allowing researchers to simultaneously compare multiple different interventions and consider their relative effects. This analytical approach is particularly useful when multiple possible interventions exist, and direct comparative studies among these interventions are limited. In the study of acupuncture treatment for PCOS-related infertility, network meta-analysis can help researchers understand the advantages and disadvantages of acupuncture compared to other treatment methods (such as medication or surgical interventions) and the differences in effectiveness among different acupuncture protocols.

In conclusion, systematic reviews and network meta-analyses of acupuncture treatment for infertility in patients with polycystic ovary syndrome (PCOS) go beyond comparing interventions, but rather provide a comprehensive evaluation of the overall effectiveness of acupuncture treatment and its relative effects compared to other treatment methods. Such studies contribute to providing more comprehensive and reliable evidence support for clinical practice.

This study also has the following limitations: Due to constraints, this study did not include the retrieval of randomized controlled trial (RCT) literature on acupuncture in languages such as French, German, and Japanese, which are included in the national language of medical insurance. This may introduce selection bias in the literature search.

The overall quality of the included studies is low, and the risk of publication bias is unclear, which is insufficient to draw definite conclusions about the efficacy of acupuncture in treating female infertility. Secondly, there is a large amount of heterogeneity in the subgroup analysis of indicators such as FSH, LH, E2, and T, which lowers the credibility of the effect size results for these outcomes. In addition, there are significant differences in acupuncture methods between different studies, which is determined by the nature of acupuncture itself. The potential publication bias inherent in studies favoring positive results may limit the reliability of the data in this study. Although this study differentiated acupuncture treatment methods, it did not address the selection of acupuncture points, needle depth, stimulation intensity, dosage, treatment duration, and course of treatment. The study only considered the common factor of stimulation produced by needling, which may introduce information bias.

## CONCLUSION

For PCOS infertility patients, acupuncture, as a complementary treatment to CC and LE, holds advantages in improving reproductive hormone levels and enhancing pregnancy success rates. The highest probability of achieving the best pregnancy outcomes is associated with the treatment combination of CC with WA and LE with MA.

## Figures and Tables

**Fig. (1):**
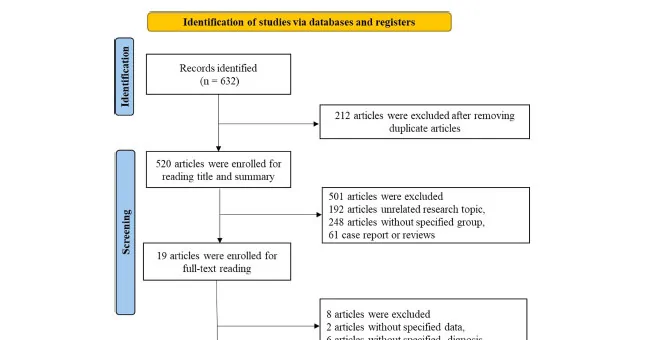
Flowchart of literature selection process.

**Fig. (2):**
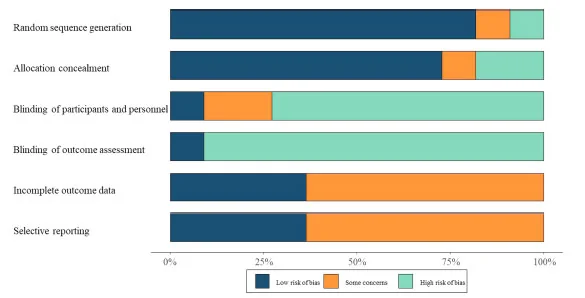
Quality assessment of included studies.

**Fig. (3):**
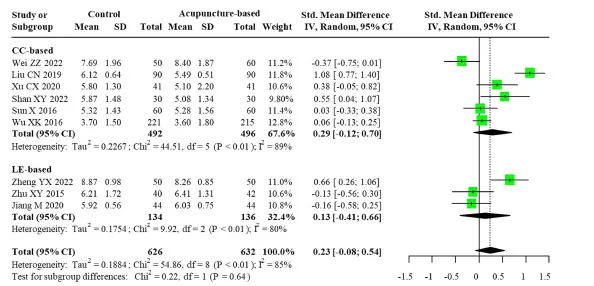
Forest plot of FSH for different treatment modalities.

**Fig. (4):**
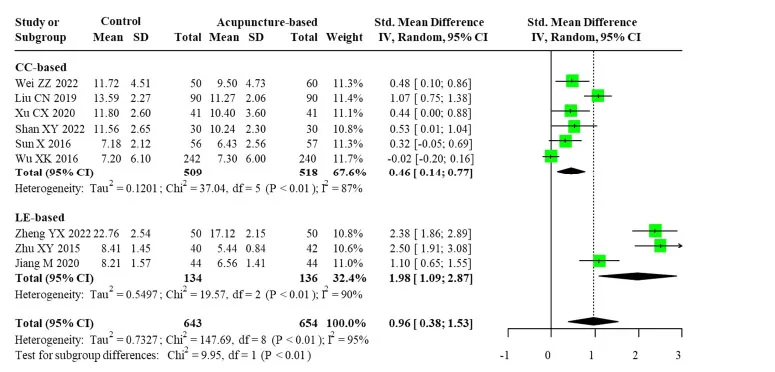
Forest plot of LH for different treatment modalities.

**Fig. (5):**
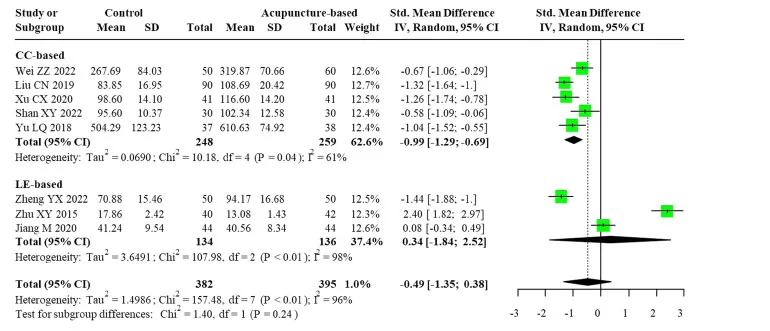
Forest plot of E2 for different treatment modalities.

**Fig. (6):**
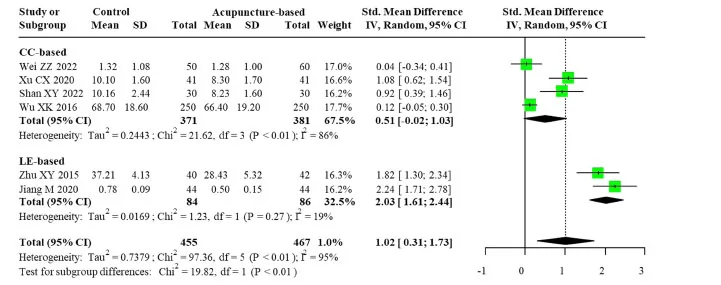
Forest plot of T for different treatment modalities.

**Fig. (7):**
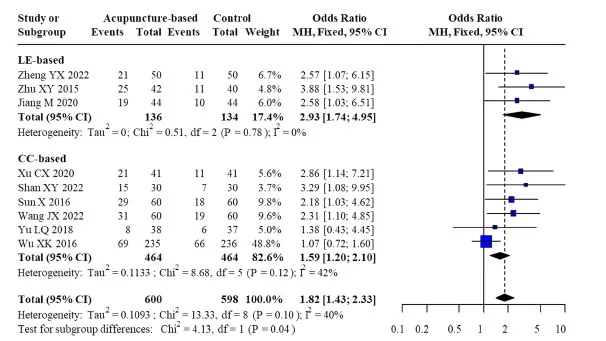
Forest plot of pregnancy outcome for different treatment modalities.

**Fig. (8):**
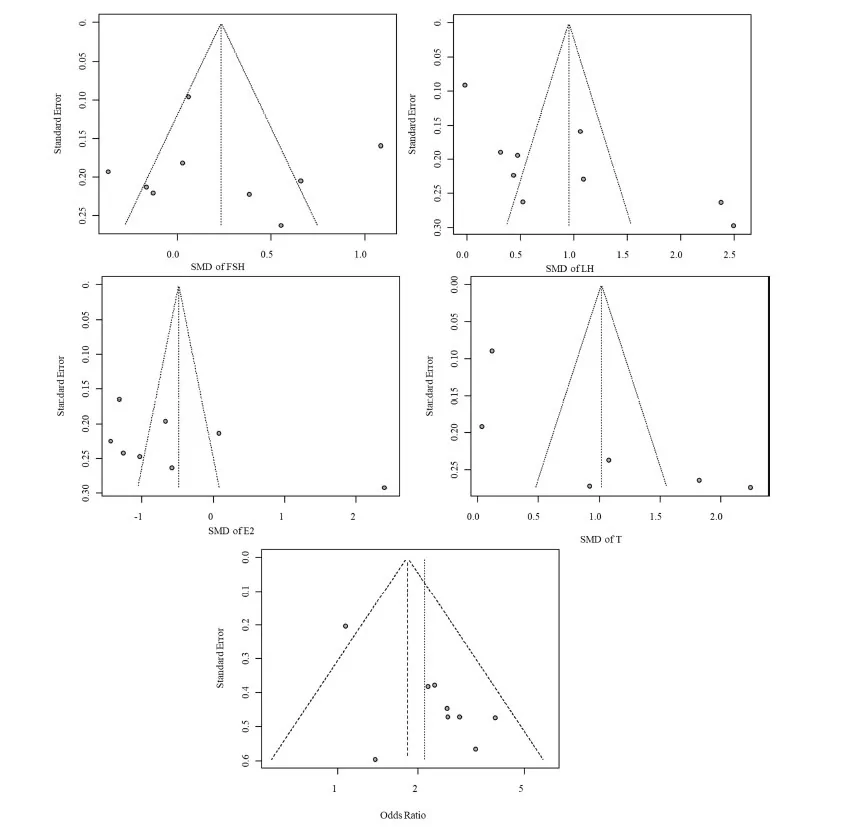
Funnel plot for publication bias among studies.

**Fig. (9):**
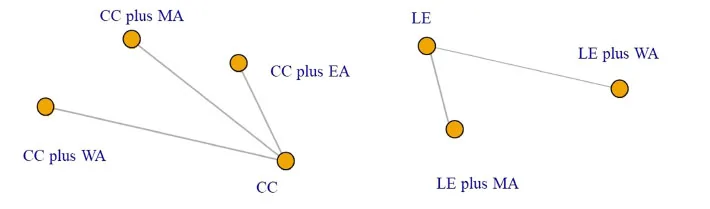
Network relationship diagram among different treatment modalities.

**Table 1:** PubMed literature search strategy.

**Search**	**Query**	**Results**
#1	“Polycystic ovary syndrome”[Title/Abstract]	16, 754
#2	“Polycystic ovarian syndrome”[Title/Abstract]	3, 845
#3	“PCOS”[Title/Abstract]	15, 565
#4	#1 or #2 or # 3	20, 717
#5	“Needling”[Title/Abstract]	9,979
#6	“Electroacupuncture”[Title/Abstract]	6, 814
#7	“Acupuncture”[Title/Abstract]	27, 586
#8	“Moxibustion”[Title/Abstract]	3, 702
#9	#5 or #6 or #7 or #8	34, 631
#10	#4 and #9	245

**Table 2:** Basic information of included studies.

**-**	**N (c)**	**N (e)**	**Age (c)**	**Age (e)**	**Intervention (c)**	**Intervention (e)**	**Course**	**Acupuncture Frequency**
Wei (2022) [14]	50	60	30.01±2.45	29.32±2.90	CC	CC+WA	3 months	Once every other day, take a single menstrual cycle as a course of treatment, until the next menstrual period to stop acupuncture, acupuncture is prohibited during menstruation.
Liu (2019) [15]	90	90	28.03±5.82	27.78±5.96	CC	CC+WA	6 months	Once every other day, take a single menstrual cycle as a course of treatment, until the next menstrual period to stop acupuncture, acupuncture is prohibited during menstruation
Zheng (2022) [16]	50	50	28.49±3.69	28.62±4.17	LE	LE+WA	3 months	Once every other day, take a single menstrual cycle as a course of treatment, until the next menstrual period to stop acupuncture, acupuncture is prohibited during menstruation
Xu (2020) [17]	41	41	28.16±4.39	27.73±5.29	CC	CC+WA	3 months	Once every other day, take a single menstrual cycle as a course of treatment, until the next menstrual period to stop acupuncture, acupuncture is prohibited during menstruation.
Zhu (2015) [18]	40	42	29.02±3.48	28.86±4.31	LE	LE+MA	3 months	Acupuncture was performed in the follicular period of the patient, that is, 4-8 days after menopause, and acupuncture was performed with electric acupuncture on 9-17 days after menstruation.
Jiang (2020) [19]	44	44	31.22±3.19	30.12±2.89	LE	LE+MA	6 months	Acupuncture was performed in the follicular period of the patient, that is, 4-8 days after menopause, and acupuncture was performed with electric acupuncture on 9-17 days after menstruation.
Shan (2022) [20]	30	30	29.50±4.11	29.40±4.25	CC	CC+WA	6 months	Acupuncture was performed in the follicular period of the patient, that is, 4-8 days after menopause, and acupuncture was performed with electric acupuncture on 9-17 days after menstruation.
Sun (2016) [21]	60	60	27.68±3.28	28.25±2.56	CC	CC+EA	3 months	Treatment started from the 5th day of menstruation or withdrawal of drug bleeding, once every other day, 3 times a week, continuous acupuncture until vaginal ultrasound detection of follicle diameter ≥18 mm, 1 menstrual cycle or 1 month as a course of treatment.
Wang (2022) [22]	60	60	31.5±3.2	30.6±3.2	CC	CC+MA	6 months	Acupuncture was performed in the follicular period of the patient, that is, 4-8 days after menopause, and acupuncture was performed with electric acupuncture on 9-17 days after menstruation.
Yu (2018) [23]	37	38	29±5	30±4	CC	CC+EA	3 months	Treatment started from the 5th day of menstruation or withdrawal of drug bleeding, once every other day, 3 times a week, continuous acupuncture until vaginal ultrasound detection of follicle diameter ≥18 mm, 1 menstrual cycle or 1 month as a course of treatment.
Wu (2016) [24]	250	250	27.8±3.4	28.2±3.4	CC	CC+MA	6 months	Acupuncture was performed in the follicular period of the patient, that is, 4-8 days after menopause, and acupuncture was performed with electric acupuncture on 9-17 days after menstruation.

## Data Availability

The data and supportive information are available within the article.

## LIST OF ABBREVIATIONS

CC: Clomiphene Citrate
CNKI: China National Knowledge Infrastructure
E2: Estradiol
EA: Electroacupuncture
FSH: Follicle-stimulating Hormone
LE: Letrozole
LH: Luteinizing Hormone
MA: Manual Acupuncture
PCOS: Polycystic Ovary Syndrome
RCT: Randomized Controlled Trials
T: Testosterone
WA: Warm Acupuncture

## STANDARDS OF REPORTING

PRISMA guidelines and methodology were followed.
